# Extracellular vesicle approach to major psychiatric disorders

**DOI:** 10.1007/s00406-022-01497-3

**Published:** 2022-10-27

**Authors:** Mojtaba Oraki Kohshour, Sergi Papiol, Ivana Delalle, Moritz J. Rossner, Thomas G. Schulze

**Affiliations:** 1 Institute of Psychiatric Phenomics and Genomics (IPPG), University Hospital, LMU Munich, 80336 Munich, Germany; 2 Department of Immunology, Faculty of Medicine, Ahvaz Jundishapur University of Medical Sciences, Ahvaz, Iran; 3 Department of Psychiatry and Psychotherapy, University Hospital, LMU Munich, 80336 Munich, Germany; 4 Department of Pathology and Laboratory Medicine, Neuropathology Service, Rhode Island Hospital, Lifespan Academic Medical Center, The Warren Alpert Medical School of Brown University, Providence, RI 02903 USA; 5 Department of Pathology and Laboratory Medicine, Boston University School of Medicine, 670 Albany Street, Boston, MA 02118 USA; 6 Department of Psychiatry and Behavioral Sciences, SUNY Upstate Medical University, Syracuse, NY USA; 7 Department of Psychiatry and Behavioral Sciences, Johns Hopkins University School of Medicine, Baltimore, MD USA

**Keywords:** Extracellular vesicles, Biomarker, Schizophrenia, Bipolar disorder, Major depressive disorder, Technical limitations

## Abstract

Over the last few years, extracellular vesicles (EVs) have received increasing attention as potential non-invasive diagnostic and therapeutic biomarkers for various diseases. The interest in EVs is related to their structure and content, as well as to their changing cargo in response to different stimuli. One of the potential areas of use of EVs as biomarkers is the central nervous system (CNS), in particular the brain, because EVs can cross the blood–brain barrier, exist also in peripheral tissues and have a diverse cargo. Thus, they may represent “liquid biopsies” of the CNS that can reflect brain pathophysiology without the need for invasive surgical procedures. Overall, few studies to date have examined EVs in neuropsychiatric disorders, and the present evidence appears to lack reproducibility. This situation might be due to a variety of technical obstacles related to working with EVs, such as the use of different isolation strategies, which results in non-uniform vesicular and molecular outputs. Multi-omics approaches and improvements in the standardization of isolation procedures will allow highly pure EV fractions to be obtained in which the molecular cargo, particularly microRNAs and proteins, can be identified and accurately quantified. Eventually, these advances will enable researchers to decipher disease-relevant molecular signatures of the brain-derived EVs involved in synaptic plasticity, neuronal development, neuro-immune communication, and other related pathways. This narrative review summarizes the findings of studies on EVs in major psychiatric disorders, particularly in the field of biomarkers, and discusses the respective therapeutic potential of EVs.

## Introduction

**Fig. 1:**
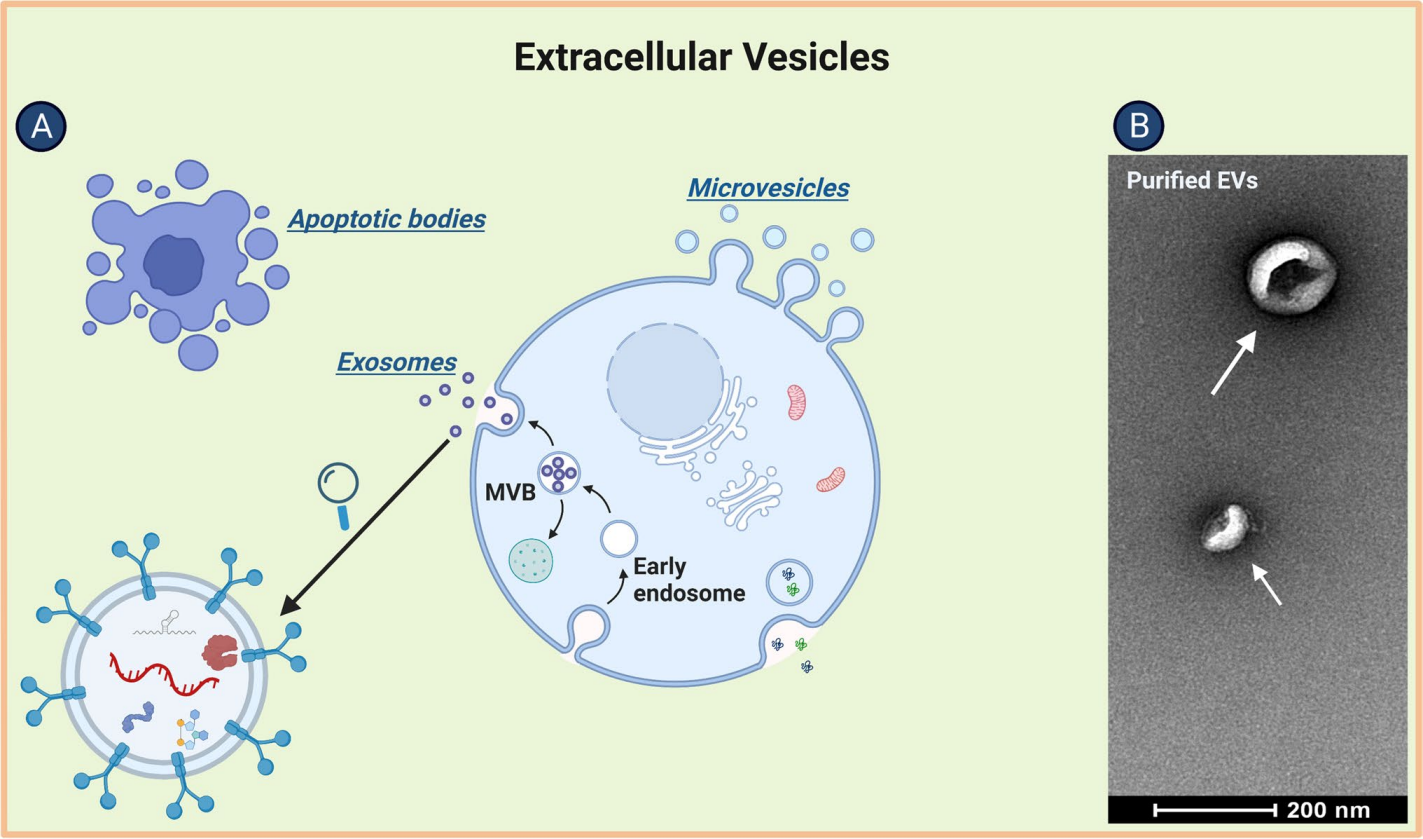
**a** Schematic diagram showing how cells release three types of extracellular vesicles: exosomes (30–150 nm), micro-vesicles (50–1000 nm), and apoptotic bodies (500–2000 nm). **b** Transmission electron microscope image of isolated and purified extracellular vesicles. (Adapted from “Extracellular Vesicle Separation by Density Gradient Ultracentrifugation,” by BioRender.com (2022). Retrieved from https://app.biorender.com/biorender-templates)

Extracellular vesicles (EVs) are nanoparticles derived from endosomes (in which case they are referred to as exosomes) or the plasma membrane (in which case they are referred to as ectosome or micro-vesicles) and are secreted from most cell types [1]. They are released via the cell membrane, either directly or after multi-vesicular bodies (MVBs) merge with the membrane (Fig. 1). Currently, there is a general consensus on EV nomenclature [1]: They can be divided into exosomes and ectosomes, depending on their biogenesis mechanism, or into small EVs (sEVs) and medium and/or large EVs (m/lEVs), depending on their size (diameter of sEVs, < 100 nm or < 200 nm; diameter of m/lEVs, > 200 nm). Because none of the reviewed publications discussed here proves that the EVs are indeed of endocytic origin (i.e., are exosomes), we use the term "EV" throughout the text [1].

EVs are present in various bodily fluids, including serum, cerebral spinal fluid (CSF), urine, saliva, and amniotic fluid, and appear to play a role in a range of biological functions, including cellular communication and maintenance mechanisms, waste removal from cells, immune responses stimulation, tissue repair and regeneration, and tumor progression [2–4]. In the central nervous system (CNS), EVs can be secreted in huge numbers by neurons, microglia, astrocytes, and oligodendrocytes and are involved in neuron–neuron and glial–neuron communication. They also participate in neurite growth; regulation of myelination and stress and immune responses; and modulation of synaptic plasticity and neuronal survival [2, 5].

**Fig. 2:**
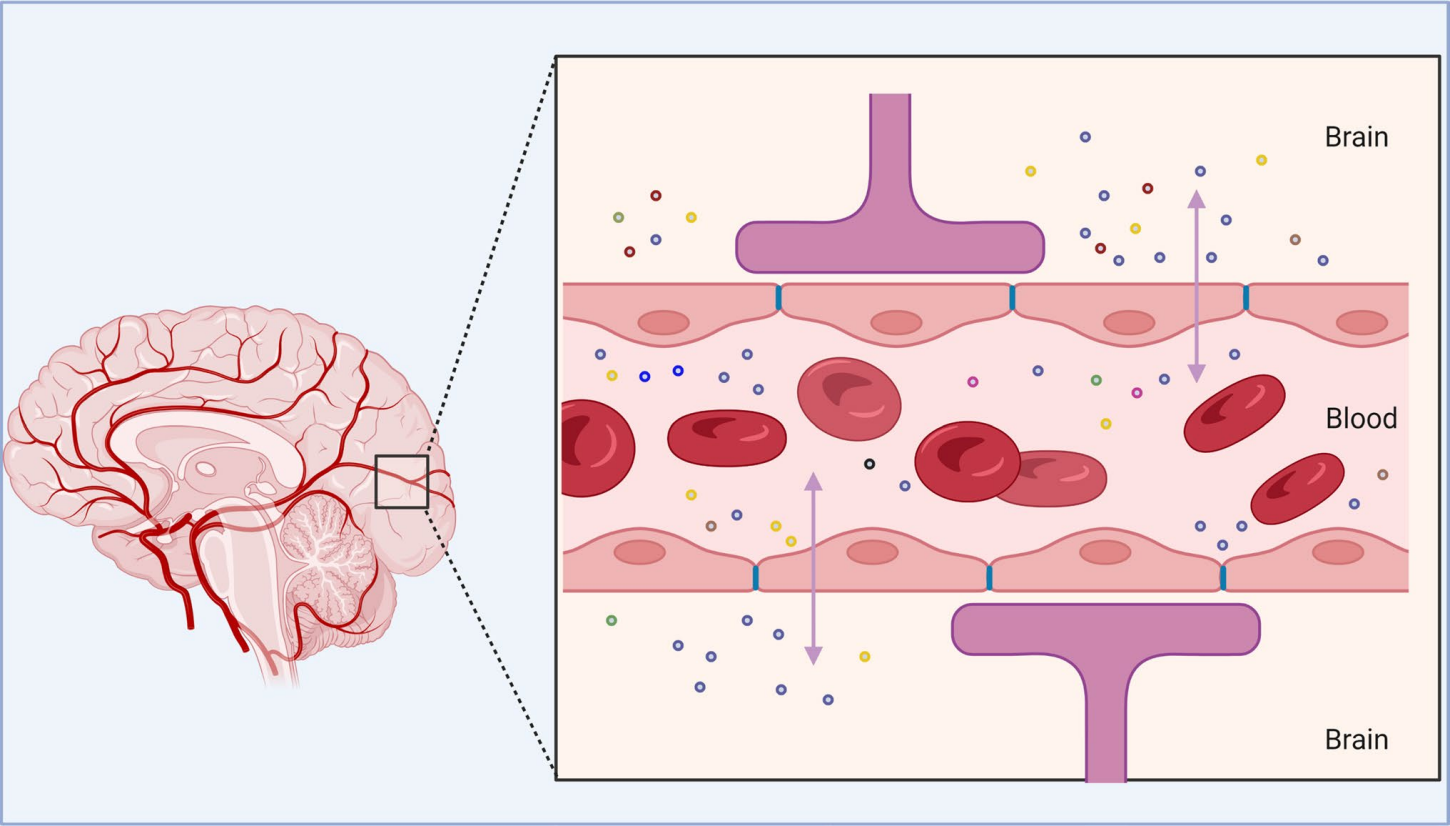
Ability of extracellular vesicles to cross the blood–brain barrier in both directions. (Adapted from “Blood Brain Barrier (Simple Longitudinal Zoom),” by BioRender.com (2022). Retrieved from https://app.biorender.com/biorender-templates)

EVs contain various bioactive compounds, such as proteins, lipids, mRNAs, microRNAs (miRNAs), other non-coding RNAs, metabolites, and DNA. Because of the diversity of their cargo and their ability to cross the blood–brain barrier (BBB) in both directions and diffuse from the site of release, they hold great potential as circulating and non-invasive biomarkers for screening, diagnostics, and treatment in complex brain disorders [4, 6]. The content of EVs may be influenced by disease status. Therefore, in CNS pathologies, their cargo may modify intercellular communication and tissue homeostasis by modulating transcription, neurogenesis, synaptic plasticity, neuronal circuit development, and neuroinflammation [7, 8]. EVs are seen as a promising type of biosignature and as a means of communication between the brain and other organs and tissues [3] (Fig. 2). Because of their effects on brain cell development and function, EVs have been hailed as an intriguing mechanism of cell-to-cell communication, with significant implications for the onset and progression of neurodegenerative and neuropsychiatric disorders [9, 10].

### Major psychiatric disorders

In 2019, mental illnesses were the second and seventh leading causes of years lived with disability and disability-adjusted life-years, respectively, worldwide [11]. The most prevalent and severe mental illnesses are schizophrenia (SCZ), bipolar disorder (BD), and major depressive disorder (MDD). All three have a polygenic architecture and partially overlapping genetic and phenotypic features; furthermore, they lack a pathophysiological signature, which represents a fundamental hurdle to their characterization and differentiation [12, 13].

SCZ is a severe, debilitating, incurable, and lifelong psychiatric illness that affects cognitive, behavioral, and emotional functioning and is characterized by symptoms, such as disorganized thinking, hallucinations, and anhedonia. The disease occurs in around 1% of the world population and is one of the top 25 causes of disability globally [14]. BD is characterized by repeated manic, hypomanic, and depressive episodes; it has a lifetime prevalence of around 1%, is associated with a high lifetime risk of suicide and is one of the top ten leading causes of disease burden for people aged 15 to 44 [13]. MDD, the most prevalent neuropsychiatric disorder in the general population (300 million cases globally), is linked to a low quality of life, functional impairment, and morbidity. Globally, it is one of the most significant causes of disability and the leading cause of suicide and is expected to rank as the second most common cause of global disease burden by 2030 [15, 16].

### Biomarker potential of EVs in brain disorders

**Fig. 3:**
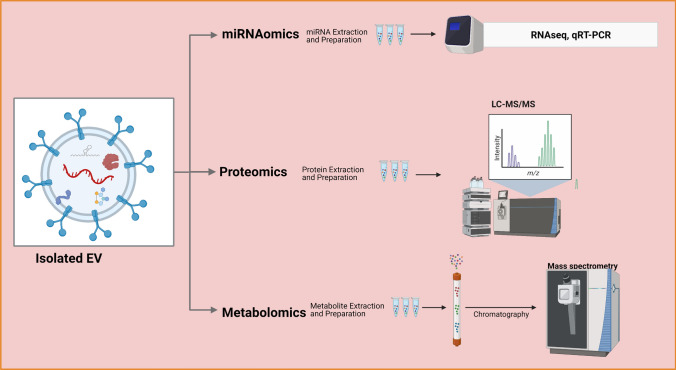
Current -omics approaches to analyzing cargos of extracellular vesicles. (Created with BioRender.com). LC–MS/MS, liquid chromatography-tandem mass spectrometry; miRNA, microRNA; qRT-PCR, quantitative real-time polymerase chain reaction; RNA-seq, RNA sequencing

In recent years, multi-omics profiling approaches have been performed in large samples of psychiatric patients with the aim to uncover disease-specific molecular fingerprints, stratify patient subgroups, predict treatment efficacy and develop drug discovery processes [17, 18]. Within this biomarker framework, first steps have been taken to use EVs to identify relevant biomarkers in SCZ, BD, and MDD (Fig. 3), and EVs have been proposed as a novel source of biomarkers and a window into brain cells and their environment [19, 20]

In vitro and in vivo experiments in mouse models have shown that EVs may mediate transmission of neurotoxic protein aggregates between neurons, and consequently, they have been referred to as “Trojan horses” [21]. In addition, they may offer a way to eliminate harmful proteins from the brain [22]. Brain cells are thought to communicate with one another or with other bodily cells by direct cell–cell interactions and soluble mediators, and researchers have suggested that EVs, and specifically their miRNA cargo, may be involved in this communication by providing a finely tuned regulatory system [23]. Changes in the function or composition of EVs from each major type of brain cells may play an important role in neuropsychiatric disorders [22].

This narrative review gives an overview of studies with the aim to provide an update on the EV approach to major psychiatric disorders.

## Methods

We performed literature searches in PubMed and included all articles published by the end of February 2022. The searches were limited to articles written in English and used the following search strings: (i) “schizophren* OR bipolar disorder OR major depressi* AND extracellular vesicle” (99 hits) and (ii) “schizophren* OR bipolar disorder OR major depressi* AND exosom*” (57 hits). After merging and curating the results, we identified 37 duplicate publications and 50 publications that were not related to the topic of interest or were the type of review articles that we did not wish to include. Ultimately, we included 69 publications in our review.

## Results

### RNA cargo

Among the various types of EV cargo, miRNA has attracted the most attention in the search for reliable and feasible biomarkers for the diagnosis and prognosis of SCZ, BD, and MDD [10, 24]. MiRNAs are significant functional components of EVs and can affect recipient cells by negatively regulating gene expression and acting as ligands at cell receptors [15, 25]. Many miRNAs are expressed in the human brain—neuronal miRNAs account for roughly 70% of all miRNAs in the body [26]—and are involved in the regulation of neuronal development and differentiation [27]. Changes in the miRNA content of EVs could affect transcriptional regulation and neurotransmitter release and function in various brain regions and may eventually contribute to the pathogenesis of major psychiatric disorders, making them potential biomarkers [10, 16, 28–30].

### Animal studies

Transplanting serum-derived EVs from patients with SCZ into mice resulted in SCZ-like behavioral and molecular phenotypes, pointing to the importance of EVs in the etiology of this disorder [10]. A comparison of bulk RNA sequencing in the hippocampus and prefrontal cortex (PFC) between mice administered EVs from either patients with SCZ or healthy controls (HCs) showed significant differences in 1887 mRNAs (999 downregulated; 888 upregulated) in the hippocampus samples and 2267 mRNAs (1130 downregulated; 1137 upregulated) in the PFC samples. The genes were significantly enriched in strongly SCZ-associated pathways, such as synaptic transmission, neurodevelopment, and behavior [10].

Peripheral injection of blood-derived EVs from HCs into chronic unpredictable mild stress (CUMS) mice reduced depression-like behaviors, suggesting that EVs have a role in depression pathophysiology [31]. Compared with control mice, CUMS mice showed significantly higher blood levels of EV-derived miR-139-5p. In cell culture assays, overexpression of miR-139-5p decreased the proliferation of neural stem cells and their differentiation into neurons [31].

EVs may be associated with the brain-derived neurotrophic factor (BDNF)/TrkB pathway and may mirror alterations in specific brain regions. Expression levels of BDNF, TrkB, and SYNAPTOTAGMIN 1 in serum-derived EVs were lower in CUMS rats than in controls and were reversed by fluoxetine [32]. Twenty-five of the 152 dysregulated serum EV miRNAs among the three groups (Control + Vehicle, CUMS + Vehicle, and CUMS + Fluoxetine) were identified as being involved in neuroplasticity and the stress response. The MAPK, Wnt, and mTOR pathways are the most notable target pathways for the miRNAs differentially expressed in CUMS rats [32]. In a rat model of depression, miRNA content of EVs from hippocampi and whole brains showed upregulation of miR-29c in hippocampal EVs and downregulation of miR-149 and miR-29c in whole-brain EVs [28]. BDNF, a well-known neurotrophin, is essential for nervous system development and maintenance [29].

### Human studies

#### Schizophrenia

MiRNA assay of PFC-derived EVs revealed significantly higher miR-497 expression in patients with SCZ than in controls [27]. MiR-497 is member of the miR-15/107 gene family, which has been linked to the development of neurodegenerative disorders [27]. Olanzapine treatment resulted in increased DJ-1 and decreased miR203a-3p levels and stopped SCZ-induced overexpression of miR203a-3p in plasma-derived EVs of patients with first-episode SCZ [33]. DJ-1 is a redox-sensitive protein, and research has suggested that miR-203a-3p can be considered as an important mediator of oxidative stress in SCZ because it targets the 3'-UTR of DJ-1 mRNA [33].

Oxidative stress in early psychosis patients (EPP) led to an increase in blood levels of the EV-derived miR-137 and a decrease in the cytochrome c oxidase subunit 6A2 (COX6A2) and changed mitophagy markers. Higher miR-137 and lower COX6A2 levels were associated with a reduction of auditory steady-state response (ASSR) gamma oscillations in an electroencephalogram [34]. Because ASSR relies on the proper functioning of networks related to parvalbumin interneurons, changes in miR-137/COX6A2 plasma EV levels could be used as a proxy marker for dysfunction of the parvalbumin interneuron cortical microcircuit, which is implicated in the psychopathology and cognitive deficits of SCZ [34].

The results of the first genome-wide miRNA expression profiling of serum-derived EVs from patients with SCZ revealed differential expression profiles that may be useful for diagnosing the disease [29]. The top differentially expressed miRNA, miR-206, was shown to influence the expression of BDNF, and miR-206 upregulation was suggested to lead to BDNF malfunction in SCZ [29].

Levels of EV-secreted miRNA-223 were increased in the orbitofrontal cortex of post-mortem brain samples from patients with SCZ and patients with BD with psychosis, and an inverse association was observed with the expression of the targets of miRNA-223, *GRIN2B* and *GRIA2* [9]. The addition of astrocytic EVs to neuronal cultures revealed similar results [9]. Treatment with the antipsychotics olanzapine and haloperidol led to cell-specific regulation of cellular and EV-derived miRNA-233, which regulates neuronal gene expression [9].

The advancement of sequencing technology has led to the discovery of a growing number of human circular RNAs (circRNAs) [35]. These non-coding RNAs can affect gene expression and regulation by acting as miRNA sponges and inhibiting gene silencing, and EV circRNAs, which are linked to early brain development, may represent the amount of circRNAs expressed in cells and tissues [36]. Analyses of plasma-derived EV circRNAs from patients with SCZ revealed the importance of four circRNAs; three of their target miRNAs, miR-34a, miR-34c, and miR-449a, are thought to play a role in the pathogenesis of SCZ [35].

#### Bipolar disorder

MiRNA assay of PFC-derived EVs revealed significantly higher miR-29c expression in patients with BD than in controls [27]. MiR-29c is induced by Wnt signaling, which is inhibited by GSK-3, a known target of lithium (the first-line treatment for BD) [27].

The miRNA content of EVs from the anterior cingulate cortex (BA24) of patients with BD showed higher levels of miR-149 in post-mortem brain tissue of patients than in that of HCs [28]. Downregulated miR-484, miR-652–3p, and miR-142–3p and upregulated miR-185–5p were shown in plasma-derived EV miRNAs of patients with BD compared with HCs [37].

#### Major depressive disorder

Expression profiling of genome-wide miRNAs in blood-derived EVs of drug-free patients with MDD and HCs identified miR-139-5p as one of the top differentially expressed miRNAs (area under the curve [AUC], 0.857; good performance) [31]. In addition, miR-139-5p showed good performance in differentiating between MDD and SCZ (AUC, 0.85). The receiver operating characteristics (ROC) curves for a cluster of 10 miRNAs that contributed most to the differentiation between MDD and HC showed excellent performance (AUC, 0.94) in differentiating between MDD and SCZ [31]. Levels of the blood-derived EV miR-139-5p were higher in patients with MDD than in HCs, indicating that miR-139-5p may be a biomarker for MDD [38].

Serum-derived EV miRNA expression profiles of negatively regulating Toll-like receptor 4 (TLR4) signaling, including let-7e, miR-21-5p, miR-145, miR-146a, and miR-155, may be useful for determining whether serum-derived EV miRNAs can be used to predict antidepressant response in MDD [39].

A study on plasma EV miRNAs from patients with treatment-resistant depression identified statistically significant differences in two miRNAs: miR-335 and miR-1292. KEGG analysis of these miRNAs identified the MAPK, Ras, and PI3K-AKT signaling pathways [30].

### Protein cargo

EVs have a variety of proteins on the surface and inside, and identifying these proteins could provide important information about the molecular mechanisms associated with their roles in EV biogenesis, structure, trafficking, immunogenicity, mediated synaptic plasticity, and cell targeting in physiological and pathological situations [8]. Thus, proteome analysis of EVs should play a key role in the development of less invasive diagnostic and therapeutic approaches for brain disorders [8].

#### Schizophrenia

Glucose hypometabolism, systemic insulin resistance (IR), and insulin signaling abnormalities in neuronal cells have been implicated in the pathophysiology of major psychiatric disorders [40–43]. Regarding individual neuronal IR biomarkers, the insulin signal transduction proteins AKT, pGSK3β, and more specifically p70S6K in plasma neuron-derived EVs (NDEVs) showed important group differences between patients with SCZ and controls: Lower neuronal IR biomarker scores were associated with higher brain glucose levels and poorer performance on verbal learning in SCZ [42].

Results from measurements of insulin signal transduction proteins in lysed NDEVs from plasma of drug-naive patients with first-episode SCZ indicated a trend for lower pS312-IRS-1 levels, decreased phosphorylated/total protein ratios of downstream serine-threonine kinases (AKT, GSK3β, mTOR, p70S6K), and lower phosphorylation ratios of mTOR in patients compared with controls, indicating reduced pathway activation [43].

Glial fibrillary acidic protein (GFAP) and α-II-SPECTRIN are two specific cargo proteins of EVs [5]. Elevated EV GFAP may be associated with increased neuro-inflammation and astrocytosis in patients with SCZ, and lower levels of EV α-II-SPECTRIN in SCZ may indicate neuronal loss [5].

According to the mild neuro-inflammation hypothesis of SCZ [which involves hypofunction of glutamatergic signaling via N-methyl-D-aspartate receptors (NMDARs) and hyperactivation of dopamine D2 receptors (D2Rs)] and triplet puzzle theory (in which the code created by the triplet amino acid homologies may help guide the receptors to each other), EV-mediated volume transmission (VT) from glial networks to neuronal networks involving distinct cytokine and chemokine receptors and their agonist ligands can result in the formation of dysfunctional and distinct glutamatergic signaling via NMDAR and D2R heteroreceptor complexes. In such heteroreceptor complexes, new allosteric receptor–receptor interactions created by the effects of agonist ligands may lead to pathological changes in D2R and NMDAR signaling, which may contribute to SCZ-like symptoms [44]. VT enables communication between all cells of the brain by diffusion in the extracellular fluid and flow in the CSF of neurotransmitters, neuromodulators, and trophic factors, where they act as VT signals and enable information handling and trophic intercellular communication, including neuron–glia and glia–glia interactions [45].

Disruption in astrocytes, which are involved in synthesis of neurotransmitters and insulation and maintenance of neural networks, can be considered as a pathophysiological mechanism in SCZ [46]. Aβ aggregates can form plaques, which cause microglial cell activation, inflammation, and neurodegeneration, and plasma levels of amyloid-beta 1–42 (ADEV-Aβ42) from astrocyte-derived extracellular vesicle (ADEVs) were higher in people with SCZ than in HCs [46].

Mitochondrial dysfunction could be one of the most important pathogenic pathways in brain disorders [47, 48]. In patients with first-episode SCZ, mitochondrial ATP production is significantly reduced, as are levels of several proteins required for mitochondrial integrity, immobilization, and endogenous mitochondrial neuroprotection [48]. Mitochondrial ATP synthase activity in ADEVs and the levels of several structurally and functionally important mitochondrial proteins in ADEVs and NDEVs are altered in patients with first-episode SCZ, so plasma NDEVs and ADEVs were hypothesized to alter brain cell metabolism by transferring mitochondrial protein abnormalities [48]. Patients with first-episode SCZ have remarkably low levels of brain mitochondrial proteins with neuroprotective and metabolic regulating functions [48].

#### Bipolar disorder

Assessments of neuronal insulin signaling at its first node, insulin receptor substrate-1 (IRS-1), and along the canonical (AKT, GSK-3β, and p70S6K) and alternative (ERK1/2, JNK, and p38-MAPK) pathways showed that NDEV biomarkers of the insulin signaling pathway were associated with cognitive dysfunction and brain structural abnormalities in patients with BD [41].

An assessment of the tumor necrosis factor-alpha (TNF-α) receptor/nuclear factor-kappa B (TNFR/NF-κB) neuro-inflammatory pathway that measured NDEV biomarkers in plasma samples of infliximab-treated patients with BD revealed that the antidepressant response to infliximab, an antagonist of TNF-α, was modulated by changes in NDEV TNFR1 levels and showed an association between lower TNFR1 levels in NDEVs and higher overall cortical thickness [49]. The alterations in NDEV cargo seen after treatment with infliximab support the hypothesis that brain insulin signaling is a pathophysiological mechanism involved in BD [41].

#### Major depressive disorder

Neuro-inflammation is thought to play a role in the pathogenesis of MDD [50]. In the brain, IL34 is secreted by neurons and astrocytes and affects microglial differentiation; when neurons are damaged, high levels of IL34 are produced, resulting in microglial dysfunction and consequent neuro-inflammation and impaired neurogenesis and synaptic plasticity [50]. Increased IL34/CD81 in NDEVs in peripheral blood has been recommended as a biomarker [50].

Baseline BDNF levels in serum-derived EVs were lower in patients with MDD than in controls, and pro-BDNF levels were higher [51]. Furthermore, levels of both BDNF and pro-BDNF in patient serum and EVs changed in the respective opposite direction after antidepressant treatment. EVs in the CNS may aid in the passage of BDNF across the BBB and be involved in modulating BDNF [51].

Serpin Family F Member 1 (SERPINF1), a secreted glycoprotein with a neuroprotective function, was the top differentially expressed protein identified in an analysis of plasma-derived EV proteins in patients with MDD and HCs [52]. SERPINF1 is a target of miR-186-5p, which is elevated in the blood of patients with MDD and suppresses SERPINF1 in the hippocampus of the CUMS mouse model [52].

Brain-enriched EVs have a membrane surface marker, L1 cell adhesion molecule (L1CAM). The mean concentration of IRS-1 in L1CAM + EVs was higher in patients with MDD than in HCs, indicating IRS-1 enrichment and increased turnover; these processes lead to decreased insulin receptor binding sensitivity and corresponding deficits in insulin signaling transduction, key molecular mechanisms of IR [40].

Vascular endothelial growth factor (VEGF) has been linked to the pathophysiology of stress-related psychiatric disorders, and plasma levels of ADEVs were positively correlated with soluble VEGF_121_ and soluble VEGF_total_ levels in patients with stress-induced exhaustion disorder (SED) [53]. More BBB leakage of ADEVs occurs in patients with SED or MDD than in HCs [53, 54].

Measurement of 14 neuron functional mitochondrial proteins in plasma NDEVs of patients with MDD revealed that levels of 11 proteins involved in different processes, such as energy generation, metabolic regulation, and mitochondrial biogenesis, were significantly lower in patients than in HCs [47].

### Metabolite cargo

EV-derived metabolites are particularly likely to represent brain pathology because of the unique features of EVs, such as their ability to cross the BBB. Dysregulation of EV-derived metabolites has been proposed to have a role in the pathophysiology of mental illness, and these metabolites were suggested as potential biomarkers for the diagnosis and evaluation of treatment approaches in psychiatric disorders [55, 56].

A panel of 25 serum EV-derived metabolites with good to excellent performance in differentiating patients with SCZ from HCs has been identified [55]. The differentially identified metabolites were shown to have significant KEGG pathway enrichment in pathways linked to glycero-phospholipid metabolism and the production of phenylalanine, tyrosine, and tryptophan. The results from metabolite gene interaction analysis revealed that four out of 264 SCZ risk genes (identified by genome-wide association studies) and two of the differentially expressed metabolites (l-arginine and taurine) are functionally related: L-arginine is associated with nitric oxide synthase 1 (*NOS1*), dipeptidase 2 (*DPEP2*), 5-aminolevulinic acid synthase 1 (*ALAS1*), and glutamic acid decarboxylase 1 (*GAD1*), and taurine is associated with *GAD1* [55].

A set of 15 serum EV-derived metabolites (chenodeoxycholic acid, lysoPE 18:0, lysoPE 14:0, N-acetylmethionine, 13-oxoODE, glycine, 1-naphthylacetic acid, 2-aminoethanesulfonic acid, D-2-aminobutyric acid, lysoPC 18:0, lysoPC 20:1, biopterin, phosphoric acid, glucosamine, and PAF C-16) was identified as a potential metabolite biomarker of BD that showed good to excellent performance in different sample sets [56]. According to the KEGG database, these differentially expressed metabolites were enriched in galactose metabolism, amino sugar and nucleotide sugar metabolism, and pentose and glucuronate interconversion pathways, indicating that impairments in sugar metabolism may have a role in the onset or progression of BD. The ROC curve for this set of metabolites resulted in an AUC of 0.886 for discriminating between BD and SCZ and of 0.771 for discriminating between BD and MDD (i.e., good to excellent performance for both) [56]. This study once again demonstrated the importance of blood EV metabolites as powerful potential biomarkers for diagnosing brain disorders.

Table 1 lists some potential biomarkers whose role and effects have been investigated in studies on EVs in major psychiatric disorders in humans.

**Table 1:** Potential biomarkers identified in studies on human EVs in major psychiatric disorders

Author/Year	Disease	Sample size	Exosome source	Potential biomarker	Remarks
Du et al. 2019 [29]	SCZ	149 cases, 146 controls	Serum	A set of 11 miRNA (miR-206 was one of the top ones that was validated by qRT-PCR)	Blood EV miRNAs may be potentially considered as a diagnostic biomarker for SCZ
Khadimallah et al. 2021 [34]	EPP (SCZ)	138 cases, 134 controls	Plasma	miR-137 and COX6A2	Changes in miR-137/COX6A2 plasma EV levels could be used as a proxy marker for dysfunction of PVI cortical microcircuit
Tsoporis et al. 2022 [33]	SCZ	11 cases 10 controls	Plasma	miR-203a-3p, DJ-1 protein	miR-203a-3p and DJ-1 protein could be promising targets for the treatment of oxidative stress-related SCZ
Tan et al. 2021 [35]	SCZ	5 cases, 5 controls	Plasma	Four differentially expressed EV circRNAs validated by qRT-PCR (has_circ: chr3_196488683_196483770_ − 4913, has_circ: chr5_69175537_69174877_ + 660, has_circ: chr5_143057747_143054439_ + 3308, has_circ: chr6_130956499_130926605_ − 29,894)	miR-34a, miR-34c, and miR-449a, three of the targets for the four identified circRNAs, are thought to play a role in the pathogenesis of SCZ
Banigan et al. 2013 [27]	SCZ and BD	8 SCZ cases, 6 BD cases, 6 controls	PFC	miR-497 and miR-29c	Levels of miR-497 and miR-29c were significantly higher in SCZ and BD, respectively, than in controls
Amoah et al. 2020 [9]	SCZ and BD	29 SCZ cases, 26 BD cases, 25 controls	Orbito-frontal cortex	miR-223	Antipsychotics exert cell-specific regulation over miRNA-233 abundance
Choi et al. 2017 [28]	BD	4 cases, 6 controls	BA24	miR-149	Significantly higher levels of miR-149 in patients; miRNAs in EVs could be possible biomarkers for BD pathogenesis
Ceylan et al. 2020 [37]	BD	69 cases, 41 controls	Plasma	miR-484, miR-652–3p, miR-142–3p, miR-185–5p	Downregulated miR-484, miR-652–3p, and miR-142–3p; upregulated miR-185–5p
Wei et al. 2020 [31]	MDD	33 cases, 46 controls	Serum	hsa-miR-139-5p	miR-139-5p is a negative regulator of neural stem cell proliferation and neuronal differentiation
Liang et al. 2020 [38]	MDD	30 cases, 30 controls	Serum	miR-139-5p	Patients with MDD had higher blood levels of EVs miR-139-5p, which might be a biomarker for MDD
Li et al. 2021 [30]	TRD (MDD)	4 cases, 4 controls	Plasma	has-miR-335 and has-miR-1292	These miRNAs are associated with synaptic function and TRD
Hung et al. 2021 [39]	MDD	52 cases, 31 controls	Serum	let-7e, miR-21-5p, miR-223, miR-145, miR-146a, and miR-155	EV negative regulatory miRNAs are useful criteria for antidepressant therapy
Lee et al. 2020 [46]	SCZ	60 cases, 60 controls	Plasma (NDEVs and ADEVs)	ADEV-Aβ42	Higher levels of ADEV-Aβ42 in cases than in controls; EV levels of Aβ and tau may be a stronger indicator of intracellular pathology than peripheral levels
Goetzl et al. 2021 [48]	FP (SCZ)	10 cases, 10 controls	Plasma (NDEVs and ADEVs)	ATP synthase, MFN2, CYPD, humanin, MOTS-c, myosin VI, SNPH (mitochondrial proteins)	Lower ATP synthase activity in ADEVs of patients; lower levels of MFN2, CYPD, humanin, and MOTS-c in ADEVs and NDEVs of patients; higher levels of myosin VI in NDEVs than in ADEVs in patients and controls; higher levels of SNPH in NDEVs of patients
Ranganathan et al. 2022 [5]	SCZ	24 cases, 12 controls	Plasma	GFAP, α-II-SPECTRIN	GFAP concentration was significantly higher and α-II-Spectrin concentration was significantly lower in patients than in controls
Mansur et al. 2020; 2021 [41, 49]	BD	55 cases	Plasma (NDEVs)	TNFR/NF-κB	Biomarker potential of NDEVs in psychiatry; brain insulin signaling is a pathophysiological mechanism involved in BD
Rhee et al. 2020 [71]	BD and MDD	42 BD cases, 30 MDD cases, 36 controls	Serum (bacteria-derived EVs)	*Prevotella 2* and *Ruminococcaceae UCG-002* genera (serum microbiome composition)	Patients with MDD had significantly higher prevalence of the *Prevotella 2* and *Ruminococcaceae UCG-002* genera than either patients with BD or controls
Kuwano et al. 2018 [50]	MDD	34 cases, 34 controls	Plasma (NDEVs)	IL34	Higher IL34/CD81 was recommended as a diagnostic biomarker for MDD
Jiang et al. 2021 [52]	MDD	10 cases, 10 controls	Plasma	SERPINF1, miR-186-5p	EVs SERPINF1 may be a valid biomarker for MDD progression; miR-186-5p may be a possible therapeutic target
Nasca et al. 2021 [40]	MDD	64 cases, 29 controls	Serum (brain-enriched EVs)	L1CAM + EVs, IRS-1	The mean number of L1CAM + EVs and the mean concentration of IRS-1 in L1CAM + EVs were higher in the MDD group than in the controls
Gelle et al. 2021 [51]	MDD	42 cases, 40 controls	Serum	BDNF, pro-BDNF	EVs may be involved in modulation of BDNF
Wallensten et al. 2021 [54]	SED (MDD)	31 SED cases, 31 MDD cases, 61 controls	Plasma (ADEVs & platelet-derived EVs)	GFAP, AQP4, CD154	Concentrations of EVs co-expressing AQP4 and GFAP and of CD154-positive EVs were significantly higher in patients with MDD than in controls; BBB leakage of ADEVs may be increased in patients with MDD
Wang et al. 2021 [64]	MDD	6 cases, 6 controls	Plasma	SIG-1R	SIG-1R was significantly enriched in EVs from patients; in depression, EVs may have an antidepressant-like effect, suggesting a potential strategy for MDD treatment
Goetzl et al. 2021 [47]	MDD	20 cases, 10 controls	Plasma (NDEVs)	12 neuron functional mitochondrial proteins	11 proteins were significantly lower and one was higher in NDEVs of patients compared with controls; numerous processes involved in neuronal mitochondria are likely to be altered in MDD
Du et al. 2021 [55]	SCZ	385 cases, 332 controls	Serum	25 EV-derived metabolites with good to excellent performance	L-arginine is connected to *NOS1, DPEP2, ALAS1*, and *GAD1*; taurine is connected to *GAD1*
Du et al. 2022 [56]	BD	32 cases, 40 controls	Serum	A set of 15 metabolites as the optimal set for differentiating patients with BD from controls	The importance of blood EVs metabolites as a powerful potential biomarker for BD diagnosis

*ADEV* astrocyte-derived extracellular vesicles, *ATP* adenosine triphosphate, *BBB* blood–brain barrier, *BD* bipolar disorder, *BDNF* brain-derived neurotrophic factor, *circRNA* circular RNA, *EPP* early psychosis patients, *EV* extracellular vesicle, *FP* first-episode psychosis, *GFAP* glial fibrillary acidic protein, *MDD* major depressive disorder, *miRNA* microRNA, *NDEV* neuron-derived extracellular vesicles, *PVI* parvalbumin interneuron, *qRT-PCR* quantitative real-time polymerase chain reaction, *SCZ* schizophrenia, *SED* stress-induced exhaustion disorder, *TRD* treatment-resistant depression

### Potential therapeutic applications

As noted above, the use of EVs as a window into the brain is being studied as a potential strategy for the diagnosis and treatment of brain diseases [57]. Furthermore, analyses of extracted brain-derived EVs provide insight into the brain molecular mechanisms underlying the biological basis of neuropsychiatric disorders [50].

In certain conditions that involve specific cellular and molecular processes, such as inflammation, physiological control of these processes with the aim to alleviate their harmful consequences might be achieved through EV-mediated communications [58]. For example, the ability of electroconvulsive therapy (ECT) to cause neurons and glia to release EVs harboring harmful proteins has been hypothesized as one mechanism that explains the success of ECT in improving outcomes in treatment-resistant CNS illness [22].

The intranasal route, which is proving to be a reliable and promising approach for delivering therapeutic compounds to the brain, is an area where EVs can be directly used and where they may play a major role in the targeted delivery of medications [57, 59].

As a new strategy for treating MDD, gene engineering may cause particular ligands to be produced on EVs, allowing for targeted delivery of these EVs [59]. By applying this approach, researchers produced rabies virus glycoprotein (RVG)-circDYM EVs in human embryonic kidney 293 T cells (HEK293T cells), which they injected via the tail vein into a mouse model of chronic unpredictable stress [59]. Various experimental and behavioral analyses revealed that circDYM was transported successfully into the mouse brain via the engineered RVG-EVs and that depression-like behaviors improved in the mice through inhibition of microglial activation and remission of astrocyte dysfunction, BBB leakiness, and peripheral immune cell infiltration [59].

**Fig. 4:**
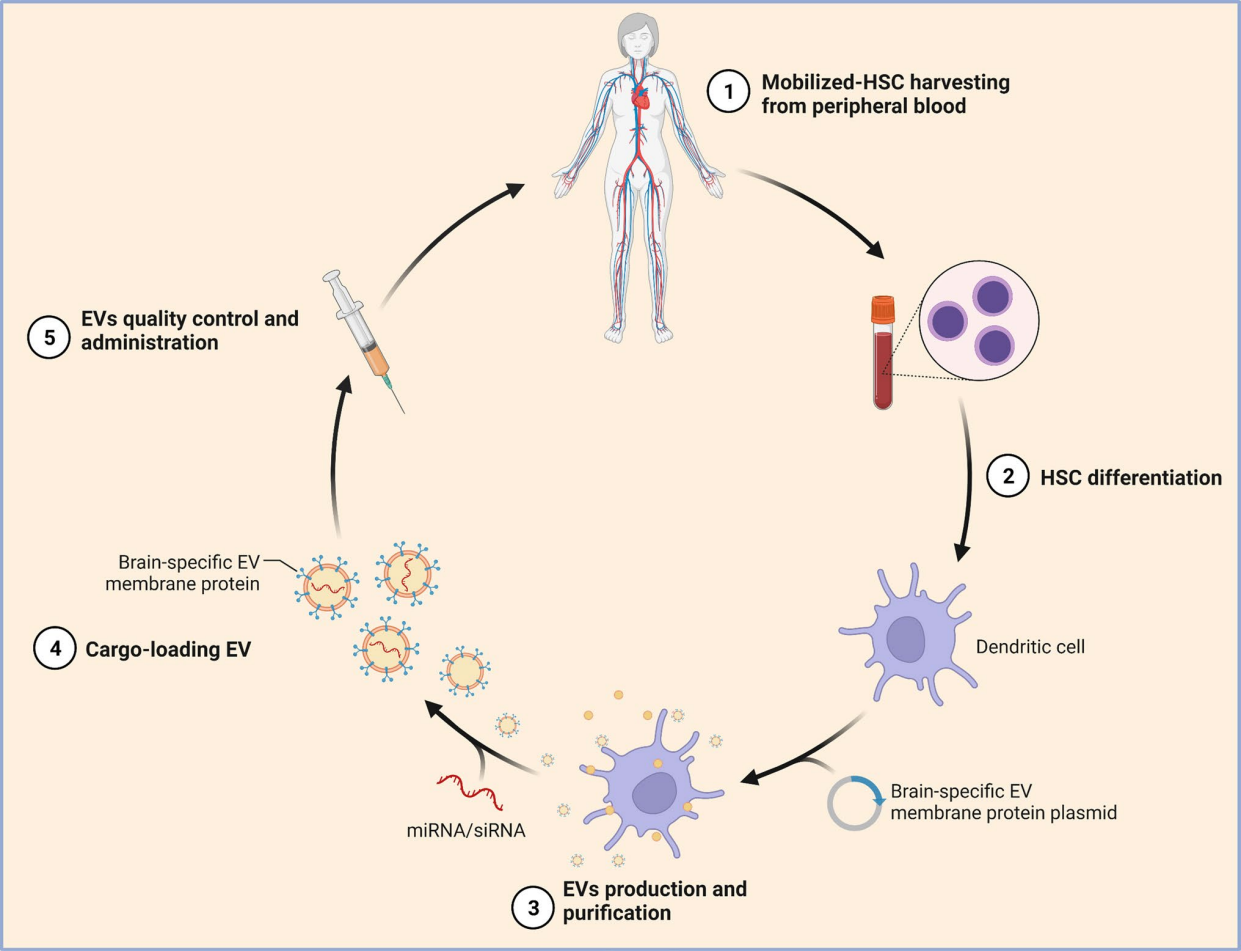
Self-derived extracellular vesicles (EVs) as a novel gene therapy. The figure shows the main steps for obtaining self-derived EVs for use as a novel gene therapy approach. EVs are capable of genetic exchange between cells and can be derived from a patient's differentiated hematopoietic stem cells and used for brain-targeted cargo delivery through expression of brain-specific peptides. By loading microRNA or small interfering RNA of the targeted gene, EVs can selectively regulate gene expression. HSC, hematopoietic stem cells; miRNA, microRNA; siRNA, small interfering RNA. (Reprinted from “Self-Derived Exosomes as a Novel Gene Therapy”, by BioRender.com (2022). Retrieved from https://app.biorender.com/biorender-templates)

EVs may be an effective system for delivering exogenous genetic material (e.g., miRNA, small interfering RNA) to recipient cells and for supporting gene therapy not only in neuropsychiatric disorders but also in many complex diseases because they are a cell-free, natural mechanism for transporting RNA between cells, they protect the RNA/gene of interest from digestion, and they are quickly taken up by the target cells [60, 61] (Fig. 4).

Several experimental studies performed in the last decade have provided evidence that mesenchymal stem cell-derived EVs (MSC-EVs) can help with the treatment of neurocognitive disorders [62]. In a study on phencyclidine (PCP)-treated mice (SCZ mouse model), intranasally injected MSC-EVs improved cognitive performance and social interaction and attenuated SCZ-like behavior by increasing the survival of gamma aminobutyric acid-producing neurons and regulating neurotransmitter activity in the CNS [63].

In patients with MDD, EVs were significantly enriched in sigma-1 receptor (SIG-1R), a promising antidepressant target that is a ligand-operated receptor chaperone. When such EVs were injected into lipopolysaccharide (LPS)-challenged mice (a depression-like model), they had beneficial effects on the increased immobility time, anhedonia-like behavior, decreased BDNF expression, and microglia activation [64]. In addition, EVs prevented LPS-induced inflammatory responses of microglial BV2 cells, and their antidepressant-like effect was inhibited when EV SIG-1R was knocked out [64]. These results support a role of EV SIG-1R in the anti-inflammatory effect of EVs in depression, suggesting a potential strategy for MDD treatment [64].

Overall, EVs hold a lot of promise for assisting the diagnosis and treatment of complex brain diseases. Advanced EV-based combinations are gaining traction in research, and more progress in solving the biological and technical difficulties in this field could enable the development of personalized EV-based treatment strategies in psychiatric disorders.

### Limitations and future perspective

EVs have emerged as a new theranostic strategy in various domains of molecular medicine because of their low immunogenicity and toxicity, biodegradability, and ability to both cross the BBB and protect their internal active components [59]. To date, EV-based medicines have not been approved by the US Food and Drug Administration in any area of medicine [65]. The successful use of EVs requires the availability of low-cost, large-scale production and high-sensitivity isolation and characterization techniques to evaluate batch-to-batch changes, as well as broadly applicable techniques for drug loading [65].

In psychiatric disorders, EVs may transform biological signals into neuroplastic modifications, leading to changes in specific brain regions and in cognitive and emotional phenotypes, and EVs may represent the missing link between biology and clinical outcome. For instance, as a non-pharmacological intervention in depressive disorders, systemic adaptation to physical exercise may cause a rapid release of EVs into the circulation, which may reduce systemic inflammation and have antidepressant effects [66, 67]. However, the isolation and characterization of EVs remains difficult because of the presence of contaminants with similar properties [68]. No well-established, universally accepted method exists for enriching solutions with total EVs or cell-specific forms of EVs from bodily fluids [69], and the separation of different EV subtypes and co-isolated protein aggregates and lipoproteins will require improvements in isolation techniques [8]. The limited quantities of CNS-derived EVs secreted by disease-relevant cells and transferred into the periphery represents another obstacle in this field [68]. Although many studies have examined EVs in various fields, especially in recent years, research is still in its early stages and is limited by both biological and technological considerations; consequently, new advancements are required [70]. In addition, because EV formation and secretion mechanisms are not completely understood, the related findings should be interpreted with caution [69]. EVs can change as diseases progress, so longitudinal studies may reveal components that better reflect alterations in brain states [69].

The use of new, creative approaches, e.g., analyzing bacteria-derived EVs from the serum microbiome in individuals with psychiatric disorders [71], or the development of a highly specific and sensitive ultra-performance liquid chromatography–tandem mass spectrometry method to detect activity of the enzyme catechol-O-methyltransferase (COMT) in EVs [72] might help to identify differentiating biomarkers. COMT participates in the metabolism of different chemical compounds, e.g., catechol drugs and catecholamine neurotransmitters, and plays a role in psychiatric disorders, and knowledge about COMT activity could be valuable for clinicians and pharmaceutical companies wanting to follow a more simplified personalized medicine approach [72].

Engineered EVs and EV-mediated drug delivery systems for targeted treatments in specific brain cells could be created by genetically or chemically changing EV membrane molecules and cargo content [73]. In EV-based gene therapy, delivery systems with EVs must become more efficient, tissue specific, and non-immunogenic [61]. Integration of EV data from different –omics outputs with other findings from neuroimaging and neuropsychological tests, as well as with genetic variations and other compounds in body fluids, has the potential to increase the precision of biomarker discovery, and the era of EV-based brain "liquid biopsy" could be approaching [5, 69].

While advances in nanotechnology and genetic engineering will aid in the delivery of desired signals to neurons and glia in the brain, EV-based methods and personalized medicine based on biological signatures in EVs have the potential to provide deep understanding of the molecular biology and physiology of behavioral alterations and treatment of brain disorders and support diagnostic, prognostic, and therapeutic approaches in psychiatry [66].

The ability of EVs to move between the CNS and peripheral circulation was recently discovered as a critical property of EVs. Numerous studies cited in our review examined peripheral EVs without any enrichment of brain cell-type specific EVs. Even though peripheral EVs may originate from organs other than the brain, examining them is still very beneficial if they act as or carry diagnostic biomarkers; however, compared with non-enriched total plasma- or serum-derived EVs, EVs enriched for neuronal origin constitute a more sensitive and reliable base for discovering biomarkers for brain disorders [68, 74]. CNS- or neuron-derived EVs in the blood have shown significant promise as "windows into the brain” that will allow alterations in brain biochemistry and intercellular communication linked to CNS diseases to be detected by peripheral blood analysis [68]. More research is needed to analyze individual, brain-derived EVs and their cargos and to create and enhance the processes that allow for the high-yield capture of these specific and high-potential EVs; such EVs may also provide information on specific molecular pathways that are directly connected to the pathophysiology of neurological disorders [68].

Unfortunately, the existing data have not been convincingly replicated, and some challenges remain in the field of EVs, as described above. Nevertheless, future advances may shed light on the precise function of EVs in the physiology and pathology of various brain disorders, such as mental illness. By improving the specific isolation and characterization of brain cell-derived EVs—such as NDEVs, ADEVs, and oligodendrocyte-derived EVs—that more directly reflect brain conditions and by applying more advanced cargo analysis technologies, research will hopefully provide relevant evidence that could be useful in defining practical EV-based diagnostic and treatment strategies for brain disorders.
